# Chemotherapy-generated cell debris stimulates colon carcinoma tumor growth *via* osteopontin

**DOI:** 10.1096/fj.201800019RR

**Published:** 2018-06-29

**Authors:** Jaimie Chang, Swati S. Bhasin, Diane R. Bielenberg, Vikas P. Sukhatme, Manoj Bhasin, Sui Huang, Mark W. Kieran, Dipak Panigrahy

**Affiliations:** * Center for Vascular Biology Research, Beth Israel Deaconess Medical Center, Harvard Medical School, Boston, Massachusetts, USA;; † Department of Pathology, Beth Israel Deaconess Medical Center, Harvard Medical School, Boston, Massachusetts, USA;; ‡ Cancer Center, Beth Israel Deaconess Medical Center, Harvard Medical School, Boston, Massachusetts, USA;; § Division of Interdisciplinary Medicine and Biology, Department of Medicine, Beth Israel Deaconess Medical Center, Harvard Medical School, Boston, Massachusetts, USA;; ¶ Vascular Biology Program, Boston Children’s Hospital, Harvard Medical School, Boston, Massachusetts, USA;; ‖ Department of Surgery, Harvard Medical School, Boston, Massachusetts, USA;; # Department of Medicine, Beth Israel Deaconess Medical Center, Harvard Medical School, Boston, Massachusetts, USA;; ** Institute for Systems Biology, Seattle, Washington, USA;; †† Division of Pediatric Oncology, Dana-Farber Cancer Institute, Harvard Medical School, Boston, Massachusetts, USA;; ‡‡ Department of Pediatric Hematology/Oncology, Boston Children’s Hospital, Harvard Medical School, Boston, Massachusetts, USA

**Keywords:** cancer, macrophage, angiogenesis

## Abstract

Colon cancer recurrence after therapy, such as 5-fluorouracil (5-FU), remains a challenge in the clinical setting. Chemotherapy reduces tumor burden by inducing cell death; however, the resulting dead tumor cells, or debris, may paradoxically stimulate angiogenesis, inflammation, and tumor growth. Here, we demonstrate that 5-FU–generated colon carcinoma debris stimulates the growth of a subthreshold inoculum of living tumor cells in subcutaneous and orthotopic models. Debris triggered the release of osteopontin (OPN) by tumor cells and host macrophages. Both coinjection of debris and systemic treatment with 5-FU increased plasma OPN levels in tumor-bearing mice. RNA expression levels of *secreted phosphoprotein 1*, the gene that encodes OPN, correlate with poor prognosis in patients with colorectal cancer and are elevated in chemotherapy-treated patients who experience tumor recurrence *vs*. no recurrence. Pharmacologic and genetic ablation of OPN inhibited debris-stimulated tumor growth. Systemic treatment with a combination of a neutralizing OPN antibody and 5-FU dramatically inhibited tumor growth. These results demonstrate a novel mechanism of tumor progression mediated by OPN released in response to chemotherapy-generated tumor cell debris. Neutralization of debris-stimulated OPN represents a potential therapeutic strategy to overcome the inherent limitation of cytotoxic therapies as a result of the generation of cell debris.—Chang, J., Bhasin, S. S., Bielenberg, D. R., Sukhatme, V. P., Bhasin, M., Huang, S., Kieran, M. W., Panigrahy, D. Chemotherapy-generated cell debris stimulates colon carcinoma tumor growth *via* osteopontin.

Whereas chemotherapy remains a mainstay in front-line cancer treatment, accumulating evidence from various animal models suggests that chemotherapy may stimulate or induce tumor initiation, growth, and metastasis (1–10); however, the mechanisms of this paradoxical chemotherapy-induced tumor growth remain poorly understood. The widely used chemotherapeutic agent, 5-fluorouracil (5-FU), is a conventional treatment for patients with multiple types of tumors, including colorectal (CRC), breast, gastric, hepatocellular, pancreatic, lung, and oral cancers (11). 5-FU reduces tumor burden by inducing cytotoxic cell death, which results in tumor cell debris (*e.g.*, apoptotic cells, necrotic cells, and cell fragments) (11). Similarly, radiation also induces apoptosis, and the resulting apoptotic tumor cells can promote tumor growth (Révész phenomenon) (12–17). Indeed, increased levels of spontaneous apoptotic cell death in the tumors of patients with cancer are associated with poor prognosis in several cancer types, including CRC, which provides clinical evidence for the Révész phenomenon (18–26). Moreover, cytotoxic therapy, such as chemotherapy and radiation, activates the apoptosis marker, caspase-3, which is associated with poor patient outcome after chemotherapy (27, 28). In one study, patients with CRC who received adjuvant 5-FU exhibited shortened survival after tumor recurrence compared with patients who had only undergone surgery (29). Taken together, tumor cell debris generated by cytotoxic therapy, such as 5-FU, may be a source of tumor growth stimulation.

We recently demonstrated that chemotherapy-generated tumor cell debris accelerates tumor growth by stimulating the production of proinflammatory cytokines by macrophages (30). Apoptotic tumor cells can stimulate macrophage chemotaxis and the production of proinflammatory cytokines by infiltrating inflammatory cells (30–33). Host responses to chemotherapy and radiation can also stimulate tumor growth by post-treatment angiogenesis (34–37). Thus, angiogenesis may be a central component in tumor stimulation *via* inflammation triggered by chemotherapy-generated cell debris. In the current study, we identified osteopontin (OPN) to be a critical mediator in the promotion of debris-stimulated tumor growth. OPN is a well-characterized protumorigenic factor that has been linked to many facets of cancer progression, including angiogenesis. OPN is often coexpressed with VEGF, and their proangiogenic activity is strongly linked in inflammatory diseases, such as cancer (38). Specifically, OPN derived from tumor stroma has been identified to mediate numerous signaling pathways that lead to tumor progression, such as cancer-associated fibroblast transformation in breast cancer (39), promotion of a stem-like phenotype in hepatocellular carcinoma (40), and activation of the PI3K (41) and NF-κB pathways (42, 43). In the clinical setting, OPN expression is linked to poor 5-yr survival in many cancer types, and the presence of both OPN and tumor-associated macrophages has been correlated with gastric cancer progression (44).

Here, we demonstrate that tumor cell debris generated by 5-FU potently stimulates tumor growth in subcutaneous and orthotopic animal models. We also show that the tumor-promoting activity of cell debris is mediated by the stimulation of macrophage and tumor cell release of the protumorigenic factor, OPN. Thus, conventional chemotherapy may contribute to tumor progression and relapse *via* tumor cell debris, the inevitable byproduct, which suggests that overcoming this dilemma between the intended induction of cell death and the tumor-promoting activity of cell debris is critical for the prevention of tumor recurrence.

## MATERIALS AND METHODS

### Cell lines

CT26 (CRL-2638) mouse colon carcinoma cells (American Type Culture Collection, Manassas, VA, USA) were cultured in RPMI-1640 medium (American Type Culture Collection) that was supplemented with 10% fetal bovine serum (FBS; Thermo Fisher Scientific, Waltham, MA, USA) and 1% l-glutamine-penicillin-streptomycin (GPS; MilliporeSigma, Burlington, MA, USA). RKO (CRL-2577) human colon carcinoma cells (American Type Culture Collection) were cultured in Eagle’s minimum essential medium (American Type Culture Collection) that was supplemented with 10% FBS and 1% GPS. RAW264.7 mouse macrophages (American Type Culture Collection) were cultured in DMEM (Thermo Fisher Scientific) that was supplemented with 10% FBS and 1% GPS. Mile Sven-1 (MS1) mouse endothelial cells (American Type Culture Collection) were cultured in DMEM that was supplemented with 5% FBS and 1% GPS. MC38 mouse colon adenocarcinoma cells (Kerafast, Boston, MA, USA) were cultured in DMEM that was supplemented with 10% FBS, 1% GPS, 0.1 mM nonessential amino acids (MilliporeSigma), 1 mM sodium pyruvate (MilliporeSigma), 10 mM Hepes (MilliporeSigma), and 50 µg/ml gentamycin sulfate (MilliporeSigma).

### Flow cytometry

Annexin V/Propidium Iodide (PI) Staining Kit (Thermo Fisher Scientific) was used according to the manufacturer’s protocol to characterize tumor cell death and analyzed by using J-Fortessa fluorescence activated cell sorting (Dana-Farber Cancer Institute; Jimmy Fund Flow Cytometry Core, Boston, MA, USA). We used FlowJo software (Treestar, Ashland, OR, USA) to quantify the results.

### Chemotherapy treatment

5-FU (MilliporeSigma) was dissolved in DMSO (MilliporeSigma). Cells were treated with 5 µM 5-FU for 72 h to generate debris. Mice were treated with 30 mg/kg 5-FU every 3 d *via* intraperitoneal injection.

### 5-FU–generated debris collection

5-FU–generated CT26, MC38, and RKO debris was prepared by refeeding 75–80% confluent T-150 flasks with 5 µM 5-FU in complete medium and incubating for 72 h at 37°C, 5% CO_2_. The resulting floating populations that contained dead cells were collected and counted by hemocytometer and centrifuged at 1250 rpm for 10 min. Supernatant (initial medium) was then aspirated, and the pelleted debris was resuspended and thoroughly washed in 10 ml of sterile PBS. Debris was then centrifuged again at 1250 rpm for 10 min. Supernatant that contained PBS with residual factors from the initial medium was aspirated, and the pelleted debris was resuspended at the final concentration in sterile PBS.

### Animal studies and approval

All animal studies were reviewed and approved by the Animal Care and Use Committee of Beth Israel Deaconess Medical Center (Boston, MA, USA; protocol 2016-070). Male mice between age 6 and 8 wk were used in these studies. Animals were housed up to 5 mice/cage in the Research North Animal Research Facility (Boston, MA, USA), a pathogen-free facility. Mice had unlimited access to sterile water and chow. Throughout each animal experiment, daily welfare evaluations were carried out per institutional committee guidelines.

### Debris-stimulated tumors

5-FU–generated CT26, MC38, and RKO debris was collected as previously described. 5-FU–generated CT26, MC38, or RKO debris (9 × 10^5^, 3 × 10^5^, or 1 × 10^5^) was prepared with a subthreshold inoculum of living cells (1 × 10^4^ CT26, 1 × 10^4^ MC38, or 2 × 10^5^ RKO) in a total volume of 100 µl sterile PBS. Cells were subcutaneously injected into the middorsum of Balb/c (The Jackson Laboratory, Bar Harbor, ME, USA), C57BL/6 (The Jackson Laboratory), or SCID (Charles River Laboratories, Wilmington, MA, USA) mice. B6.129S6(Cg)-Spp1^tm1Blh^/J mice (The Jackson Laboratory), which originated on a mixed Black Swiss, 129S6 background and were backcrossed to C57BL/6 for at least 10 generations and maintained as a homozygote, were used in OPN knockout (KO) studies. The formula (width × width × length × 0.52) was used to calculate tumor volumes.

### Orthotopic studies

Living CT26 and CT26 debris was generated and collected as described above. Balb/*c* mice were anesthetized by using 2–4% isoflurane (Patterson Vet, Devens, MA, USA). The ventral side was prepared by shaving and applying betadine. A 2-cm incision was made through the skin and peritoneum layers, and the cecum was identified and exposed. Living CT26 (1 × 10^3^) and CT26 (9 × 10^4^) debris alone or in combination were injected in a total volume of 50 µl of sterile PBS into the wall of the cecum. The incision was closed using 4-0 chromic gut sutures (MedRep Express, Hurricane, UT, USA).

### Macrophage conditioned medium

Dulbecco’s PBS with calcium and magnesium (Lonza, Basel, Switzerland) was used to adhere RAW264.7, primary mouse resident peritoneal macrophages, or monocyte-derived human macrophages to tissue culture dish. Macrophages were exposed to 5-FU–generated CT26, MC38, or RKO tumor cell debris in a 1:4 macrophage:debris ratio for 1 h after which PBS was aspirated and macrophages were washed and refed with serum-free medium. Macrophages were incubated overnight at 37°C before conditioned medium (CM) was collected. CM was centrifuged at 1100 rpm for 5 min to remove particulates and stored at −20°C.

### Primary human monocyte–derived macrophage collection

Whole blood from healthy human donors was obtained from the Boston Children’s Hospital Blood Donor Center (Boston, MA, USA.) Human peripheral blood mononuclear cells were isolated *via* Histopaque-1077 (MilliporeSigma) density gradient. Monocytes were differentiated into macrophages by using 10 µg/ml granulocyte M-CSF (R&D Systems, Minneapolis, MN, USA) in RPMI-1640 for 6 d before use.

### Primary mouse resident peritoneal macrophage collection

Mice were euthanized by cervical dislocation and their abdomens were disinfected with 70% ethanol. An incision was immediately made through the skin on the ventral side of the mouse. Chilled PBS solution (10 ml) was injected into the peritoneal cavity. After rocking the mouse, PBS in the peritoneal cavity was collected and spun at 1000 rpm for 10 min at 4°C. PBS was aspirated and the pellet was resuspended and cultured in DMEM/F12 (Thermo Fisher Scientific) medium that was supplemented with 10% FBS (Thermo Fisher Scientific).

### Mouse plasma collection

Mice were bled retro-orbitally using Micro-Hematocrit Capillary Tubes (Fisherbrand, Pittsburgh, PA, USA) into blood collection tubes with K2E (BD Biosciences, Billerica, MA, USA). Whole blood was centrifuged at 2000 *g* for 20 min at 4°C within 30 min of collection. The plasma layer was isolated and stored at −80°C.

### ELISA

Mouse and human OPN ELISAs (R&D Systems) were used to quantify the OPN concentration in CM and mouse plasma according to the recommended protocol. We used VERSA max Microplate Reader (Molecular Devices, San Jose, CA, USA) to quantify the assays.

### Neutralizing Abs

Mouse OPN Abs (R&D Systems) were used to neutralize OPN in CM at a concentration of 3 µg/ml for 1 h on ice. As a control, normal goat IgG (R&D Systems) was also used to treat CM at 3 µg/ml for 1 h on ice. *In vivo* OPN depletion was achieved with the administration of 20 µg OPN Ab/mouse every 3 d in sterile PBS *via* intraperitoneal injection. Likewise, 20 µg normal goat IgG/mouse every 3 d served as control.

### MTT assay

MS1 endothelial cells were plated at 5000 cells/well in a clear bottom 96-well plate (Corning Costar, Corning, NY, USA). Cells were refed with CM (from RAW264.7, RAW264.7 exposed to colon tumor debris, MC38, or MC38 exposed to debris) that was treated with 3 µg/ml control IgG or OPN Ab as described above. A Cell Proliferation Kit 1 (MTT; Roche, Basel, Switzerland, USA) was used to measure MS1 viability after 24 h according to the manufacturer’s protocol. We used VersaMax Microplate Reader (Molecular Devices) to quantify the assay.

### Immunohistochemistry

Tumor tissue (*n* = 4–5 mice/group) was fixed in 4% paraformaldehyde (Electron Microscopy Sciences, Hatfield, PA, USA) for 24 h, then transferred and stored in 70% ethanol. Paraffin-embedded sections were deparaffinized with xylene and rehydrated through a series of descending concentrations of ethanol. Antigen retrieval was performed by using recombinant RT-PCR grade Proteinase K (20 µg/ml; Roche). Endogenous peroxidase and proteins were blocked with 3% H_2_O_2_ and Tris-NaCl–blocking buffer, respectively. Slides were incubated with purified rat anti-mouse CD31 (BD Pharmingen, San Jose, CA, USA) overnight at 4°C. Mouse absorbed biotinylated anti-rat secondary Ab (Vector Laboratories, Burlingame, CA, USA) was used. TSA Biotin System (PerkinElmer, Waltham, MA, USA) was used for signal amplification, and the ImmPact DAB Peroxidase Substrate Kit (Vector Laboratories) was used to visualize staining. Tissues were counterstained with hematoxylin and mounted with permount (Thermo Fisher Scientific). A Zeiss A1 Scope and AxioCam ICc5 and Zeiss Efficient Navigation (ZEN) software (Zeiss, Oberkochen, Germany) was used to obtain images of slides after staining (5–10 fields/tumor). ImageJ (National Institutes of Health, Bethesda, MD, USA) was used to quantify the number of vessels per field.

### The Cancer Genome Atlas analysis of patient secreted phosphoprotein 1 gene expression

To determine the association of *secreted phosphoprotein 1 (SPP1)* with survival, we performed survival analysis using data from The Cancer Genome Atlas (TCGA). Survival analysis was performed on RNA sequencing of samples from patients with colon adenocarcinoma and rectal carcinoma (45). Expression data were divided into high, middle, and low expression groups on the basis of mean expression. Survival analysis was performed by using Kaplan-Meier (K-M) analysis from survival package in R (The R Foundation, Vienna, Austria). The K-M estimate is a nonparametric maximum likelihood estimate of the survival function that is based on the number of survivors and nonsurvivors at any given time point. Results of the survival analysis were visualized by using K-M survival curves with log rank testing. Results were considered significant if *P* values from log rank test were < 0.05.

### Gene Expression Omnibus analysis of patient SPP1 gene expression

To evaluate any association of *SPP1* gene expression and response toward leucovorin, 5-FU, oxaliplatin (FOLFOX) therapy, we obtained microarray data for 166 patients with colorectal cancer who were treated with FOLFOX. Raw data were downloaded from the Gene Expression Omnibus database (GSE81653) (46) and normalized by using the RMA algorithm to generate transcript-level data. The quality of normalized data was determined by using standard Affymetrix QC matrices and Boxplots using R/Bioconductor packages (Affymetrix, Santa Clara, CA, USA). After preprocessing and normalization, significant differentially expressed genes were identified by comparing recurrence with nonrecurrence samples after FOLFOX treatment. We implemented the Linear Model for Microarray and RNA-Seq Data (Limma), which uses moderate *T* statistics to identify significant differentially expressed genes. Genes with absolute fold change of at least 1.2 and values of *P* < 0.05 were considered significant.

### Statistical analysis

For *in vivo* experiments, 2-sided, unpaired Student’s *t* test and single-factor ANOVA analysis were used to assess differences between groups. We used Student’s *t* test to evaluate significance for *in vitro* experiments. Summary data are reported as means ± sem. In the orthotopic colon tumor model, we used Fisher’s exact test to evaluate survival differences over time. Log-rank test was used to evaluate the significance of K-M curves of patients. The *P* value of *SPP1* expression in patients with recurrence *vs.* nonrecurrence was calculated in R using Limma for moderate *T* statistics and Student’s *t* test. Values of *P* < 0.05 were considered statistically significant.

## RESULTS

### Systemic administration of 5-FU stimulates the growth of a subthreshold inoculum of tumor cells

**Figure 1:**
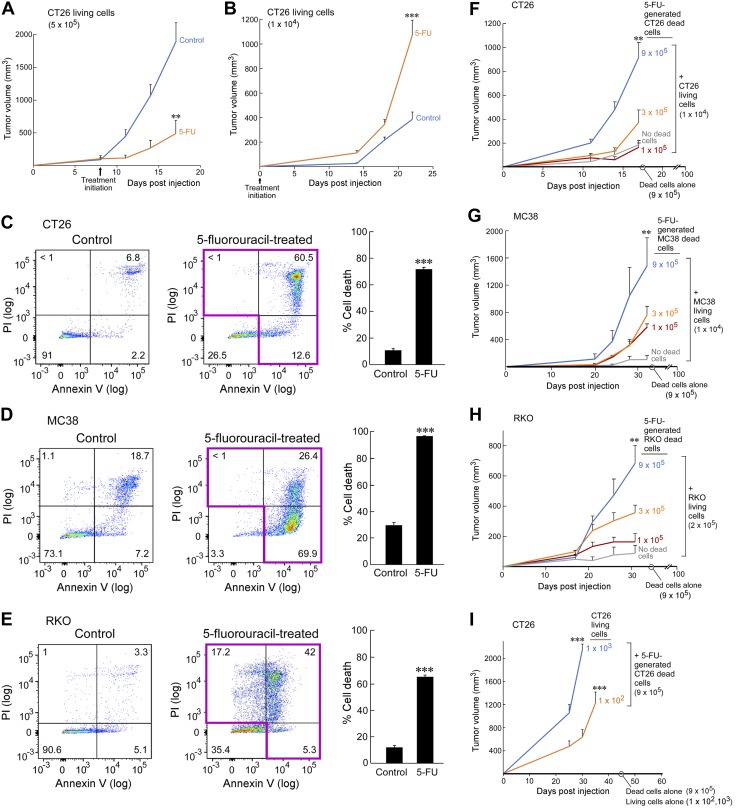
5-FU–generated colon carcinoma cell debris stimulates tumor growth. *A*) CT26 tumors (5 × 10^5^ living cells) treated systemically with 5-FU (30 mg/kg every 3 d). Treatment was initiated once tumors reached 100–200 mm^3^ (*n* = 5 mice/group). *B*) CT26 tumors (1 × 10^4^ living cells) treated systemically with 5-FU (30 mg/kg every 3 d) starting on the day of injection (*n* = 5 mice/group). *C*–*E*) Flow cytometry analysis of apoptotic (annexin V positive/PI negative; bottom right quadrant), necrotic (annexin V negative/PI positive; upper left quadrant), and late apoptotic/necrotic (annexin V positive/PI positive; upper right quadrant) cell death *via* annexin V/PI staining of whole population *in vitro* cell cultures of CT26 (*C*), MC38 (*D*), and RKO (*E*) cells that were treated with 5 µM 5-FU for 72 h *vs.* control (*n* = 3/group). *F*, *G*) Debris-stimulated CT26 (*F*) and MC38 (*G*) tumor growth from 5-FU–generated dead cells coinjected with a subthreshold inoculum of 1 × 10^4^ living cells in Balb/c and C57BL/6 mice, respectively (*n* = 5–10 mice/group). *H*) Debris-stimulated RKO tumor growth from 5-FU–generated dead cells coinjected with a subthreshold inoculum of 2 × 10^5^ living cells in SCID mice (*n* = 5 mice/group). *I*) 5-FU–generated CT26 dead cells (9 × 10^5^) coinjected with low inoculums of CT26 (1 × 10^2^ or 1 × 10^3^ living cells; *n* = 5 mice/group). Data are presented as means ± sem. Two-tailed Student’s *t* test for final tumor measurements were used throughout unless specified; 1-way ANOVA analysis was used for comparison of 3 or more groups. **P < 0.01, ***P < 0.001.

To assess the efficacy of the chemotherapeutic agent 5-FU on colon tumor growth initiated by both high and low inoculums of tumor cells, we used the CT26 mouse colon carcinoma model in Balb/*c* mice. Systemic treatment of 5-FU (30 mg/kg) inhibited the growth of established (100–200 mm^3^) CT26 colon tumors that were generated from a high inoculum of 5 × 10^5^ living cells (**Fig. 1*A***). In contrast, the same treatment initiated on the day of injection stimulated tumor outgrowth from a subthreshold inoculum—defined as a low number of tumor cells that would otherwise not generate a rapidly growing tumor—of 1 × 10^4^ living CT26 cells (Fig. 1*B*). These results indicate that 5-FU treatment can paradoxically have growth-promoting activity on small tumors. We also examined an intermediate inoculum of 1 × 10^5^ CT26 cells in both settings and found that, similarly, at tumor establishment 5-FU treatment inhibited tumor growth (Supplemental Fig. 1*A*); however, at the 1 × 10^5^ inoculum, treatment with 5-FU initiated on the day of tumor cell injection stimulated tumor growth (Supplemental Fig. 1*B*).

To examine the cytotoxic activity of 5-FU *in vivo*, we created a single-cell suspension from size-matched control tumors *vs.* established tumors that were treated with systemic 5-FU from 5 × 10^5^ CT26 cells. Using flow cytometry analysis, we observed increased cell death in tumors that were treated with 5-FU compared with size-matched control tumors (Supplemental Fig. 1*C*), thus confirming that systemic 5-FU treatment indeed induces tumor cell death *in vivo*.

### 5-FU–generated colon tumor cell debris stimulates tumor growth

To investigate the mechanism of the observed tumor-stimulatory activity of chemotherapy (Supplemental Fig. 1*B*), we determined whether this was mediated by 5-FU–generated dead tumor cells—apoptotic cells, necrotic cells, and cell fragments—hereafter referred to as debris. Accordingly, we developed 3 distinct 5-FU–generated debris-mediated models using 3 colon cancer cell lines: CT26, a mouse colon carcinoma; MC38, a mouse colon adenocarcinoma; and RKO, a human colon carcinoma. To examine the role of cell debris in tumor growth, we prepared tumor cell debris by treating tumor cells with 5 µM 5-FU *in vitro* for 72 h. Death of CT26, MC38, and RKO cells induced by 5-FU was characterized by annexin V/PI staining and the whole population was quantified by flow cytometry analysis (Fig. 1*C–E*). Cells that stained positive for annexin V (apoptotic), PI (necrotic), or annexin V and PI (late apoptotic/necrotic) are collectively defined as debris. Flow cytometry analysis also confirmed that the floating population from 5-FU treatment, which was used for all debris experiments, was approximately 100% dead for all 3 cell lines (Supplemental Fig. 1*D*).

In the CT26 mouse tumor model, 5-FU–generated CT26 debris stimulated subcuteneous growth of a subthreshold inoculum of living colon carcinoma cells in a dose-dependent manner by more than 4-fold in Balb/*c* mice. Tumor cell debris alone did not produce any visible tumors at 100 d postinjection. Conversely, increasing the amount of 5-FU–generated CT26 debris (1 × 10^5^, 3 × 10^5^, or 9 × 10^5^ dead cells) coinjected with the subthreshold inoculum of CT26 cells (1 × 10^4^ living cells) resulted in accelerated tumor growth in a dose-dependent manner (Fig. 1*F*). To ensure that stimulation of primary tumor growth by 5-FU–generated colon tumor cell debris was not strain or cell line specific, we next examined the tumor cell line MC38 in C57BL/6 mice. MC38 debris generated by 5-FU *in vitro* coinjected with a subthreshold inoculum of living MC38 (1 × 10^4^ living cells) also accelerated tumor growth in a dose-dependent manner (Fig. 1*G*). Moreover, human RKO tumor cell debris generated by 5-FU stimulated the dose-dependent xenograft growth of a subthreshold inoculum of living RKO tumor cells (2 × 10^5^) in immunocompromised SCID mice (Fig. 1*H*).

To further evaluate the potency of debris in triggering outgrowth from the subthreshold inoculum of tumor cells, we titrated the number of living CT26 cells down to 1 × 10^3^ or 1 × 10^2^ coinjected with a fixed quantity of 5-FU–generated tumor cell debris (9 × 10^5^). We found that CT26 alone (1 × 10^2^ or 1 × 10^3^ living cells) did not result in macroscopic tumors, even at 60 d postinjection; however, CT26 debris generated *in vitro* by treatment with 5-FU promoted tumor growth of an inoculum as low as 1 × 10^2^ living tumor cells (Fig. 1*I*).

To determine whether debris-stimulated colon tumor growth was specific to subcutaneous tumors, we also established a debris-stimulated orthotopic colon tumor model in which 1 × 10^3^ living CT26, 9 × 10^4^ 5-FU-generated CT26 debris, or the combination of dead and living cells were injected directly into the wall of the cecum in Balb/*c* mice. Remarkably, 5-FU–generated CT26 debris coinjected with living CT26 cells resulted in shortened survival and moribund mice compared with mice that were injected with either living or dead cells alone (Supplemental Fig. 1*E*). These results indicate that debris generated by chemotherapy potently stimulates the growth of tumors that otherwise would not exhibit rapid growth.

### 5-FU–generated colon tumor cell debris stimulates OPN secretion by macrophages and tumor cells

**Figure 2:**
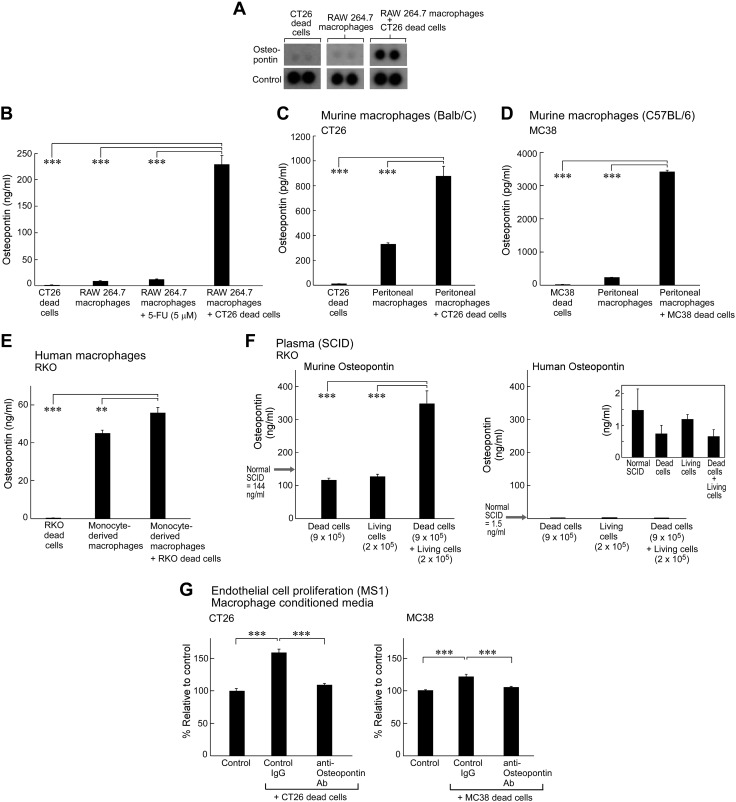
5-FU–generated colon tumor cell debris stimulates OPN secretion by macrophages and tumor cells. *A*) Angiogenic cytokine/chemokine profile of CM from RAW264.7 macrophages exposed to 5-FU–generated CT26 dead cells compared with macrophages or dead cells alone. *B*) ELISA quantification of OPN concentration in CM from RAW264.7 macrophages exposed to 5 µM 5-FU (1 h) and 5-FU–generated CT26 dead cells (1 h) compared with control macrophages or dead cells alone (*n* = 3/group). *C*, *D*) ELISA quantification of murine OPN released by primary resident peritoneal macrophages exposed to 5-FU–generated CT26 (*C*) or MC38 (*D*) dead cells *vs.* macrophages or dead cells alone; macrophages isolated from Balb/*c* or C57BL/6 mice, respectively (*n* = 3/group). *E*) ELISA quantification of human OPN released from primary human monocyte-derived macrophages exposed to 5-FU–generated RKO dead cells *vs.* macrophages or dead cells alone (*n* = 3/group). *F*) ELISA quantification of murine OPN (left) and human OPN (right) in plasma from SCID mice injected with debris-stimulated tumors (9 × 10^5^ 5-FU–generated RKO dead cells alone, 2 × 10^5^ living RKO alone, or 2 × 10^5^ living RKO coinjected with 9 × 10^5^ 5-FU–genererated RKO dead cells; *n* = 4–5/group). *G*) Viability of MS1 mouse ECs treated with CM from RAW264.7 macrophages pretreated with control, 3 µg IgG/ml, or 3 µg anti-OPN Ab/ml and exposed to 5-FU–generated CT26 (left) or MC38 (right) dead cells (*n* = 12/group). Data are presented as means ± sem. Two-tailed Student’s *t* test was used throughout unless specified. **P < 0.01, ***P < 0.001.

As proangiogenic cytokines released by activated immune cells in tumor stroma are known to mediate tumor-promoting activity (47), we next measured the extent to which debris stimulates the release of proangiogenic cytokines by macrophages. An angiogenic protein array of 53 cytokines revealed that conditioned medium (CM) from RAW264.7 macrophages cocultured with 5-FU–generated CT26 debris contained increased levels of OPN compared with CM from unstimulated macrophages or CT26 debris alone (**Fig. 2*A***). Quantification of OPN levels in CM by ELISA demonstrated that the release of OPN by debris-stimulated macrophages was increased ∼10-fold compared with dead cells alone, unstimulated macrophages, or 5-FU–treated macrophages, thereby excluding the possibility that increased OPN secretion was a result of OPN or retained 5-FU that may have been released from the debris (Fig. 2*B*). To ensure that the release of OPN was not specific to the RAW264.7 cell line, we next collected primary resident macrophages from the peritoneum of mice. Similarly, 5-FU–generated CT26 or MC38 debris also stimulated OPN release by primary resident mouse peritoneal macrophages that were isolated from Balb/*c* or C57BL/6 mice, respectively, compared with unstimulated macrophages or debris alone (Fig. 2*C, D*). In parallel, we also examined the activity of human colon tumor cell (RKO) debris on macrophages that were differentiated from monocytes isolated from healthy human donor blood. Indeed, RKO human colon carcinoma debris generated by 5-FU also stimulated OPN secretion by primary human monocyte-derived macrophages compared with unstimulated macrophages or RKO debris alone (Fig. 2*E*).

In addition to macrophages, we also examined the production of OPN by tumor cells themselves after exposure to tumor cell debris. CM from CT26 and MC38 tumor cells that were cocultured with 5-FU–generated CT26 or MC38 debris, respectively, exhibited higher OPN levels compared with tumor cells or tumor cell debris alone (Supplemental Fig. 2*A*, *B*). Of interest, the RKO cell line did not generate human OPN, even after stimulation with debris (Supplemental Fig. 2*C*). These findings indicate that although tumor cell debris stimulates OPN secretion of tumor cells that already produce OPN, debris-stimulated tumor growth is likely not dependent on tumor-derived OPN, as RKO debris stimulates RKO tumor growth *in vivo* (Fig. 1*H*).

### Systemic 5-FU treatment and tumor cell debris increase OPN levels *in vivo*

To measure OPN levels *in vivo*, we used ELISA to quantify murine (host derived) and human (tumor cell derived) OPN concentrations in the plasma of SCID mice bearing debris-stimulated human RKO xenografts. Mice with debris-stimulated tumors exhibited drastically higher host (murine) OPN levels compared with mice that were injected with either RKO debris alone or the subthreshold inoculum of living RKO cells alone, both of which expressed OPN levels that were comparable to non–tumor-bearing control mice (Fig. 2*F*, left); however, because RKO does not express OPN (Supplemental Fig. 2*C*), human OPN was barely detected in the plasma of any of the samples (Fig. 2*F*, right). Mouse and human OPN have minimal cross-reactivity (48); therefore, elevated OPN levels in mice bearing debris-stimulated tumors are likely host derived, which suggests that the OPN stimulated by debris is generated by the tumor microenvironment.

In addition to debris-stimulated tumor growth, we also examined 1 × 10^4^ CT26 tumors that were systemically treated with 5-FU beginning on the day of tumor cell injection (Supplemental Fig. 2*D*). We quantified OPN levels in the plasma of these mice by using ELISA and found that mice that were treated with systemic 5-FU exhibited higher levels of plasma OPN compared with control (Supplemental Fig. 2*E*). To determine whether this increase in plasma OPN is dependent on the mice bearing tumors, we treated non–tumor-bearing mice with 5-FU and found no significant difference in OPN levels in the plasma of control mice compared with 5-FU–treated mice (Supplemental Fig. 2*F*), which suggests that the increase in plasma OPN is dependent on mice bearing tumors.

### 5-FU and tumor cell debris enhance endothelial cell viability and angiogenesis

To investigate a potential cellular process governed by OPN that may mediate debris-stimulated tumor growth, we determined whether debris-stimulated OPN release increases endothelial cell (EC) viability, a critical component of angiogenesis (49). CM from RAW264.7 mouse macrophages that were cocultured with 5-FU–generated CT26 or MC38 tumor cell debris increased the viability of MS1 ECs compared with CM from control macrophages. Strikingly, and consistent with our hypothesis, pretreatment of CM with a neutralizing anti-OPN Ab suppressed debris-stimulated EC viability (Fig. 2*G*), which suggests that this process is OPN dependent. In addition, we examined EC viability in response to CM from control unstimulated MC38 tumor cells and MC38 cells that were stimulated with debris and determined that CM from debris-stimulated MC38 cells increased EC viability compared with control MC38, which was inhibited by pretreatment with anti-OPN Ab (Supplemental Fig. 2*G*).

Furthermore, we assessed the activity of systemic 5-FU treatment on angiogenesis *in vivo*. After treating mice that were injected with 1 × 10^4^ CT26 with systemic 5-FU initiated on the day of tumor cell injection (Supplemental Fig. 2*D*), we stained paraffin-embedded control and 5-FU–treated tumors for the EC marker, CD31. Microvessel quantification revealed higher vessel density in tumors of mice that were treated with 5-FU compared with control (Supplemental Fig. 2*H*), which suggests that debris-stimulated tumor growth is mediated by enhanced tumor angiogenesis.

### OPN expression is associated with poor prognosis in patients with colorectal cancer

**Figure 3:**
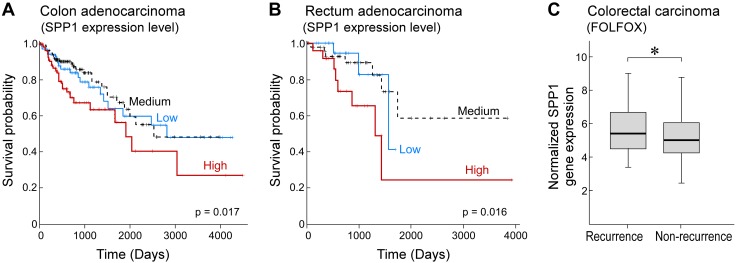
Elevated *SPP1* gene expression levels are associated with poor prognosis and tumor recurrence in patients with colorectal cancer. *A*) K-M analysis exhibiting the correlation between *SPP1* RNA expression and survival probability of patients with colon adenocarcinoma (*n* = 283 patients). *P* = 0.017. *B*) K-M analysis exhibiting the correlation between *SPP1* gene expression and survival probability of patients with rectum adenocarcinoma (*n* = 94 patients). *P* = 0.016. *C*) Gene Expression Omnibus data analysis of *SPP1* gene expression in patients with colorectal cancer who have received FOLFOX chemotherapy comparing patients with recurrence *vs.* no recurrence (*n* = 166 patients). *P* = 0.03. We used the log-rank test to evaluate the significance of K-M curves. *P* values were calculated in R using Limma for moderate *T* statistics between the 2 groups. *P < 0.05.

*SPP1* is the gene that encodes OPN. To examine OPN expression in patients with colorectal cancer, we examined samples from patients with colon adenocarcinoma and rectum adenocarcinoma using data from The Cancer Genome Atlas to analyze the correlation between expression of *SPP1* RNA and survival. We found that when patients were categorized into high, medium, and low expression of *SPP1*, there was a strong negative correlation between *SPP1* expression levels and survival. In both populations of patients with colon adenocarcinoma (**Fig. 3*A***) and rectum adenocarcinoma (Fig. 3*B*), higher SPP1 expression was correlated with shortened survival compared with patients with medium and low *SPP1* expression.

We also examined *SPP1* gene expression in patients with colorectal cancer who have received FOLFOX therapy, a commonly used cocktail of 3 agents (leucovorin, 5-FU, and oxaliplatin). The association between *SPP1* expression and recurrence status was evaluated in 166 patients with colorectal cancer who were treated with FOLFOX. Analysis indicated that *SPP1* expression was significantly elevated in patients with recurrence compared with no recurrence (fold change = 1.33; *P* = 0.03; Fig. 3*C*).

### Debris-stimulated tumor growth is OPN dependent

**Figure 4:**
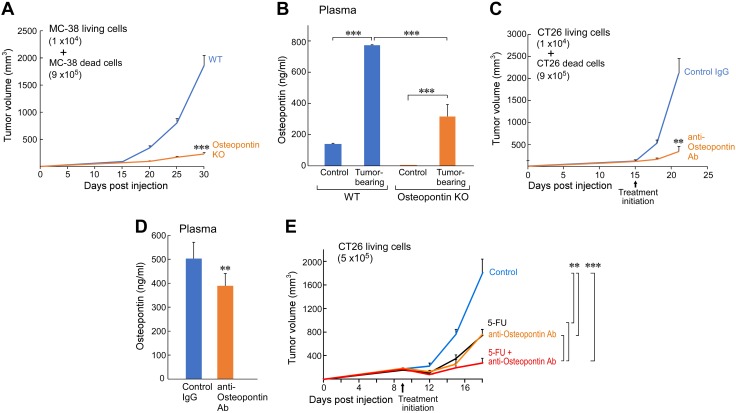
Debris-stimulated tumor growth is OPN dependent. *A*) Debris-stimulated MC38 (1 × 10^4^ living MC38 coinjected with 9 × 10^5^ 5-FU–generated MC38 tumor cell debris) tumor growth in OPN KO mice compared with WT mice (*n* = 4–5 mice/group). *B*) ELISA quantification of OPN in plasma from WT and OPN KO mice that were injected with debris-stimulated MC38 tumors or control non-tumor–bearing WT and OPN KO mice (*n* = 4–5/group). *C*) Debris-stimulated CT26 tumor growth (1 × 10^4^ living CT26 coinjected with 9 × 10^5^ CT26 tumor cell debris) treated with anti-OPN Ab or control IgG (20 µg Ab/mouse every 3 d; *n* = 5 mice/group). *D*) ELISA quantification of OPN in plasma from mice that were injected with debris-stimulated CT26 tumors treated with control IgG or anti-OPN Ab (*n* = 5 mice/group). *E*) CT26 tumors treated with 5-FU (30 mg/kg every 3 d) and/or anti-OPN Ab (20 µg/mouse every 3 d; *n* = 5 mice/group). Data are presented as means ± sem. Two-tailed Student’s *t* test for final tumor measurements were used throughout unless specified; 1-way ANOVA analysis was used for comparison of 3 or more groups. **P < 0.01, ***P < 0.001.

To determine the extent to which host-derived OPN contributes to debris-stimulated tumor growth, we generated debris-stimulated MC38 tumors (1 × 10^4^ living cells + 9 × 10^5^ dead cells) in wild-type (WT) and OPN knockout (KO) mice. In OPN KO mice, we observed an 87% reduction in debris-stimulated MC38 tumor growth compared with WT mice, which suggests that debris-stimulated tumor growth critically depends on host-derived OPN (**Fig. 4*A***). OPN in the plasma of non-tumor–bearing OPN KO mice was not detectable. Consistent with our previous findings, WT mice that were injected with debris-stimulated tumors exhibited drastically higher levels of OPN in their plasma compared with non-tumor–bearing WT mice and OPN KO mice bearing debris-stimulated tumors (Fig. 4*B*). Of interest, OPN was detected in OPN KO mice bearing debris-stimulated MC38 tumors, which suggests that tumor cells themselves are also a source of OPN. This is consistent with *in vitro* findings that MC38 produces OPN, which is further stimulated when exposed to debris (Supplemental Fig. 2*B*).

To further examine plasma OPN levels *in vivo*, WT mice were injected with 9 × 10^5^ dead cells alone, 1 × 10^4^ living cells alone, or the combination of 1 × 10^4^ living cells and 9 × 10^5^ dead cells. ELISA analysis determined that OPN levels in the plasma of mice that were injected with dead cells alone and living cells alone were not significantly different from each other; however, mice that were injected with the combination of dead and living cells exhibited drastically higher levels of plasma OPN than either mice injected with living or dead cells alone (Supplemental Fig. 3).

To confirm that the genetic deletion of OPN inhibits debris-stimulated tumor growth and to establish the clinical relevance of pharmacologic OPN modulation, we used an OPN-neutralizing Ab in the CT26 debris-stimulated tumor model. Once debris-stimulated CT26 tumors were established (100–200 mm^3^), mice were treated systemically with anti-OPN Ab or isotype control IgG. Anti-OPN Ab dramatically inhibited debris-stimulated primary CT26 growth compared with mice that were treated with the isotype control IgG (Fig. 4*C*). ELISA analysis confirmed the neutralizing activity of the anti-OPN Ab *in vivo*, as the detectable levels of OPN in the plasma of anti-OPN Ab–treated mice were reduced compared with mice that were treated with the isotype control (Fig. 4*D*). Thus, pharmacologic modulation of OPN levels recapitulated the suppression of debris-stimulated tumor growth observed in OPN KO mice.

Given the efficacy of the anti-OPN Ab for inhibiting debris-stimulated tumor growth, we next systemically administered anti-OPN Ab as adjuvant therapy to 5-FU once CT26 tumors were established (100–200 mm^3^). Tumor growth was partially suppressed (60–70%) by 5-FU treatment alone (see also Fig. 1*A*). Systemic therapy with the anti-OPN Ab in combination with 5-FU resulted in the sustained inhibition of primary tumor growth compared with either therapy alone (Fig. 4*E*). These findings suggest that targeting OPN with a pharmacologic agent could serve as a powerful adjuvant therapy by neutralizing a potential mechanism of debris-stimulated tumor growth induced by chemotherapy.

## DISCUSSION

The reported activity of tumor cell debris that results from cytotoxic therapies remains complex. Whereas studies have confirmed the Révész phenomenon and demonstrated that radiation-induced dead tumor cells have tumor promoting and proangiogenic activity (13, 16, 17, 34), others have reported anticancer activity of tumor cell debris (50–52). In the current study, we investigated mechanisms that govern the protumorigenic activity of tumor cell debris in the context of colorectal carcinoma. Our data support the concept that dead tumor cells produced by the chemotherapeutic agent, 5-FU, have potent tumor-stimulatory activity. We demonstrate that tumor cell debris accelerates tumor growth by stimulating the production of OPN by both tumor cells and cells in the host microenvironment, including macrophages.

To study the activity of tumor cell debris on tumor growth, we established a subthreshold inoculum model in mice. The implantation of a low number of tumor cells mimics dormancy or minimal (residual) growth (53). Inoculums of tumor cells for each tumor cell line were titrated to produce slow and inconsistent tumor growth when the inoculum was injected alone. This constellation mirrors the residual tumor cells that may remain post-therapy and act as a source for local recurrence (54). We found that although systemic treatment of 5-FU inhibited the growth of established tumors, it stimulated tumor growth in mice that were injected with the subthreshold inoculum. This paradoxical finding suggests that 5-FU activity depends on tumor size. 5-FU likely suppresses the growth of established tumors, but exhibits protumorigenic activity in the setting of minimal residual disease that results in 5-FU–mediated tumor recurrence. Using this subthreshold inoculum model, we demonstrated that the tumor-promoting activity of tumor cell debris is robust and dose dependent. This was confirmed in 3 independent colon carcinoma cell lines and remained consistent in 3 strains of mice—Balb/*c*, C57BL/6, and SCID. As debris-stimulated tumor growth was observed in immunocompromised SCID mice, it suggests that the tumor-stimulating activity of the debris may be independent of the adaptive immune system. As tumor recurrence rates after treatments, such as chemotherapy, remain high in patients with colorectal cancer (29), our studies suggest that the tumor cell debris that results from cytotoxic treatment may contribute to the stimulation of the growth of residual tumor cells. This is consistent with previous studies on radiation- and chemotherapy-generated debris that stimulates living tumor cells, thus acting as a feeder (13, 30). As viable tumor cells can inevitably survive during cytotoxic therapy that is aimed to kill tumor cells (54), 5-FU may be a double-edged sword.

Our results demonstrate that one mechanism by which tumor cell debris stimulates tumor growth is *via* the increase of OPN secretion by cells in the tumor microenvironment. Specifically, we focused on macrophages, which are known to express OPN (55–59) and promote tumor growth and progression *via* angiogenesis (60), as well as colon tumor cells, which can generate high levels of OPN in a cell-autonomous manner (61). We found that both macrophages and 2 of our tumor cell lines—CT26 and MC38—generated a baseline level of OPN; however, when exposed to tumor cell debris generated by 5-FU, both macrophages and tumor cells secreted drastically higher levels of OPN. Of interest, the third tumor cell line, RKO, did not secrete detectable OPN. In addition, we observed elevated levels of murine OPN in SCID mice bearing debris-stimulated human RKO tumors, but negligible levels of human OPN. As murine and human OPN have minimal cross-reactivity (48), elevated OPN levels in mice bearing debris-stimulated tumors are likely host-derived, thus suggesting that the OPN that mediates the stimulatory activity of debris is produced by the tumor microenvironment. Of importance, our studies with OPN KO mice also suggest that host-derived OPN production is critical to debris-stimulated tumor growth. OPN KO mice that were injected with debris-stimulated tumors exhibited OPN in their plasma, which confirmed that tumor cell debris stimulated tumor cell production of OPN *in vivo*. However, the increased levels of OPN in the plasma of WT mice that were injected with debris-stimulated tumors and their drastically accelerated tumor growth compared with OPN KO mice suggest that OPN produced by the host is indeed critical for debris-stimulated tumor growth.

OPN plays important roles in several hallmarks of cancer, such as angiogenesis (62), cell proliferation (63, 64), and invasion and metastasis (65). Increased levels of OPN have been observed in a number of cancer types, including colorectal, breast, lung, and ovarian (65, 66). Our independent analyses of OPN gene expression, encoded by *SPP1*, demonstrate a strong negative correlation between OPN expression levels and the survival of patients with colon adenocarcinoma and rectum adenocarcinoma. This suggests that increased OPN expression may be an indicator of poor patient prognosis and that reducing the expression or activity of OPN may have therapeutic benefit. Furthermore, analysis of *SPP1* RNA expression in patients with colorectal cancer who have received FOLFOX treatment revealed higher *SPP1* expression in patients who have experienced recurrence compared with patients with no tumor recurrence, which suggests that inhibiting *SPP1* expression may represent a novel approach by which to prevent tumor recurrence.

We also assessed the therapeutic activity of systemic anti-OPN neutralizing Ab treatment in inhibiting debris-stimulated tumor growth in mice. Whereas treatment with either 5-FU or anti-OPN Ab alone achieved tumor growth inhibition, we found that mice that were treated with a combination of 5-FU and anti-OPN Ab exhibited drastically inhibited tumor growth compared with mice that received either treatment alone. Treatment with only chemotherapy inhibited tumor growth by promoting tumor cell death, thus reducing tumor burden. Tumor growth was also inhibited with anti-OPN Ab treatment alone. As a result of extensive reports on the protumorigenic roles of OPN (38–42, 44, 57–59), it is not surprising that neutralizing OPN has some therapeutic benefits. Many tumors themselves produce OPN, and the dead tumor cells that result from natural turnover may stimulate stroma-derived OPN. Thus, OPN levels are increased even in tumor-bearing animals that do not receive chemotherapy; however, the efficacy of treatment with chemotherapy may be inherently limited because of the stimulation of OPN by dead tumor cells that inevitably result from cytotoxic treatment. Of importance, our data suggest that neutralizing the activity of OPN *via* an anti-OPN Ab may be a potent adjuvant therapy alongside chemotherapy. Furthermore, this study highlights an urgent need for novel pharmacologic agents that inhibit OPN expression or activity.

Exposing that chemotherapy can paradoxically stimulate tumor progression has pathophysiologic and clinical relevance in a wide variety of cancers and provides a new rationale for combination therapy. Our findings indicate that exploiting debris-stimulated OPN represents a novel pharmacologic target for adjuvant treatment to chemotherapy. Whereas generation of tumor cell debris throughout treatment may be an inherent therapeutic limit to current cytotoxic cancer therapies, inhibiting OPN or other mediators of the tumor-promoting activity of cell debris, in combination with cytotoxic treatment, may represent a novel approach to preventing therapy-induced tumor growth and recurrence.

## Supplementary Material

This article includes supplemental data. Please visit *http://www.fasebj.org* to obtain this information.

## Abbreviations

5-FU: 5-fluorouracil
CM: conditioned medium
CRC: colorectal cancer
EC: endothelial cell
FBS: fetal bovine serum
FOLFOX: leucovorin, 5-fluorouracil, oxaliplatin
GPS: l-glutamine-penicillin-streptomycin
K-M: Kaplan-Meier
KO: knockout
MS1: Mile Sven-1
OPN: osteopontin
PI: propidium iodide
SPP1: secreted phosphoprotein 1
WT: wild type
