# Supplementary figures and images for: Chemotherapy-generated cell debris stimulates colon carcinoma tumor growth via osteopontin
Source: FASEB J. 2018 Jun 29;33(1):114–25. doi: 10.1096/fj.201800019RR (PMC6355061; doi:10.1096/fj.201800019RR)

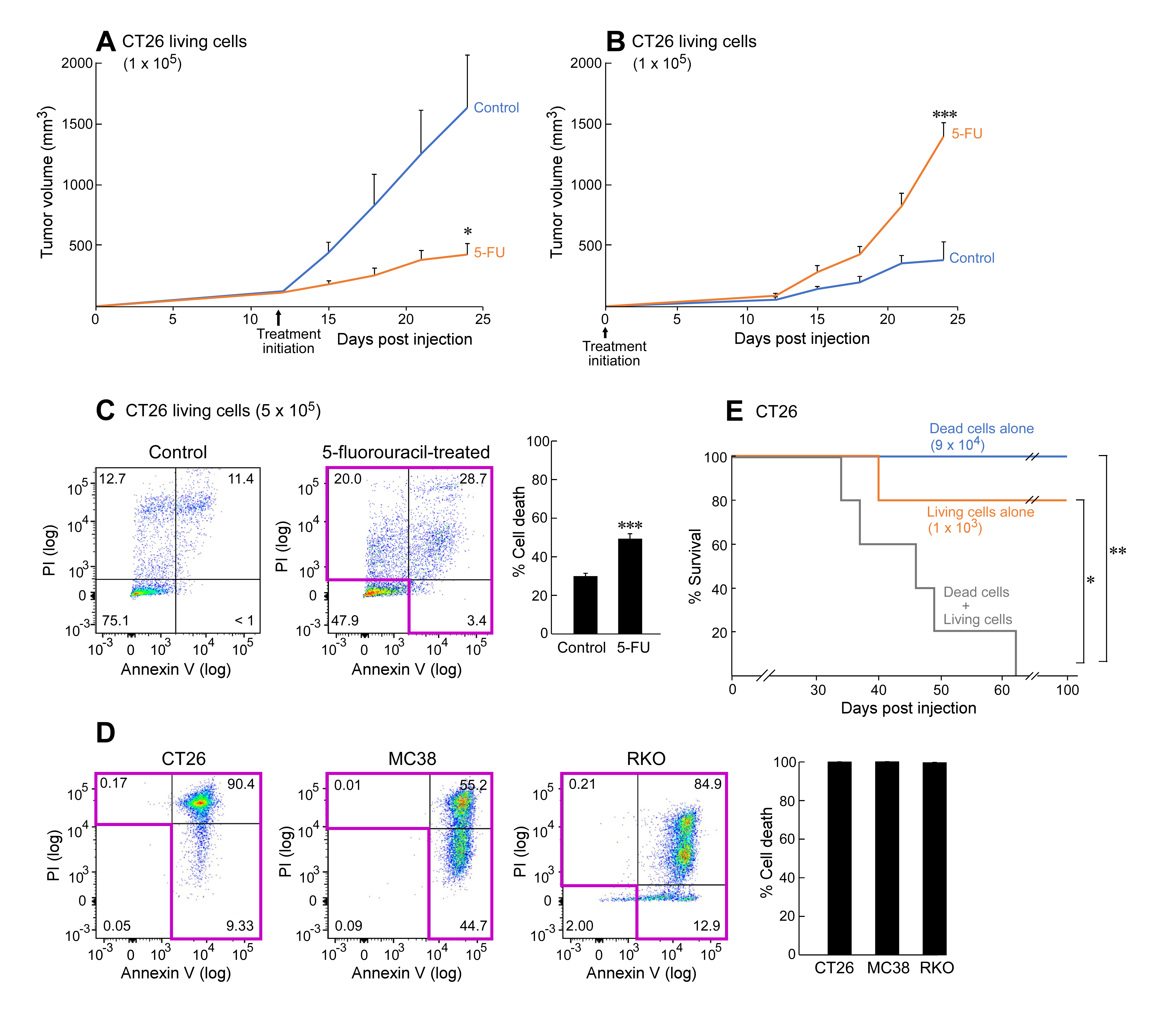

Supplement: Supplementary file 1 [file fj.201800019RR.sf1.tif]

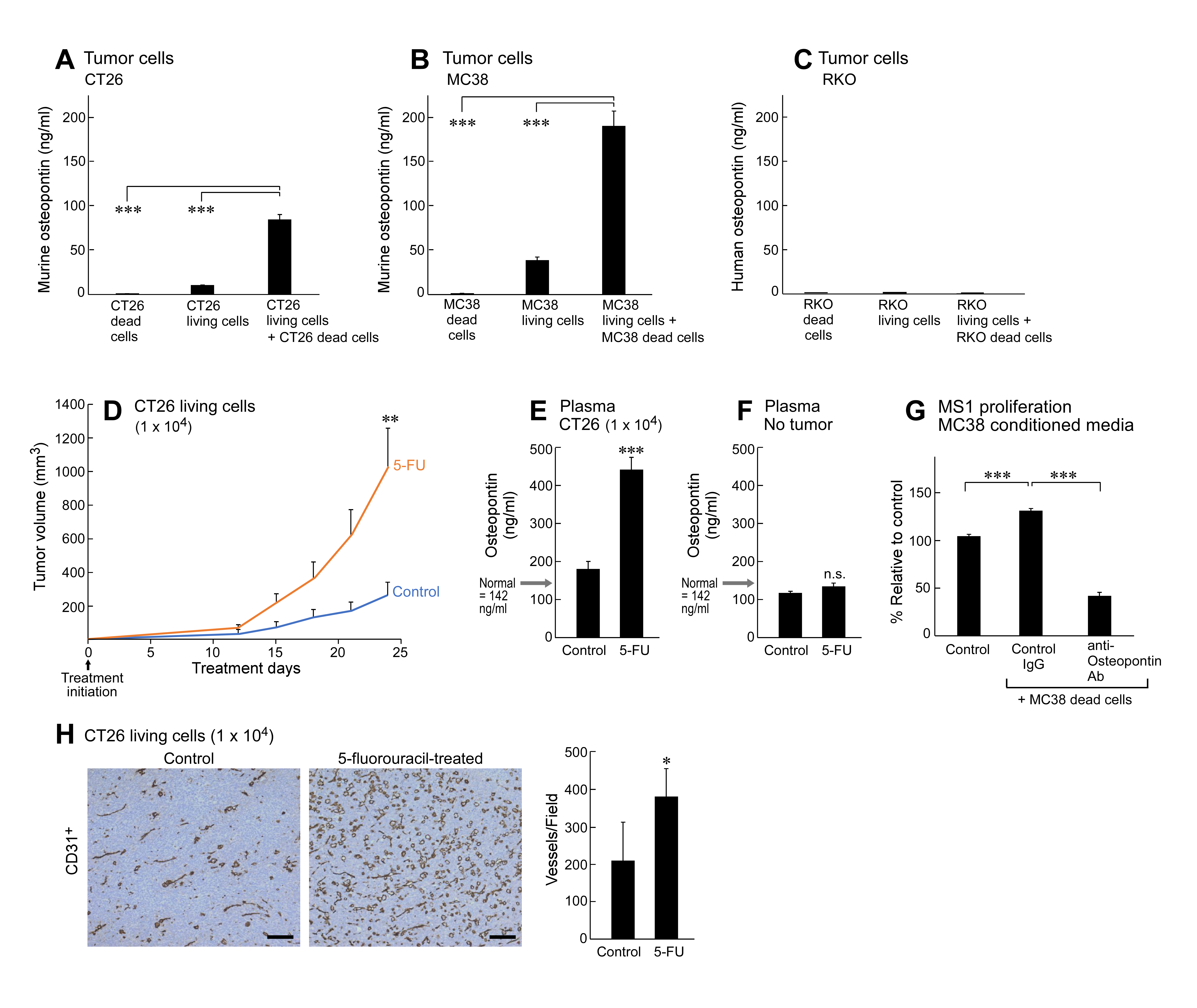

Supplement: Supplementary file 2 [file fj.201800019RR.sf2.tif]

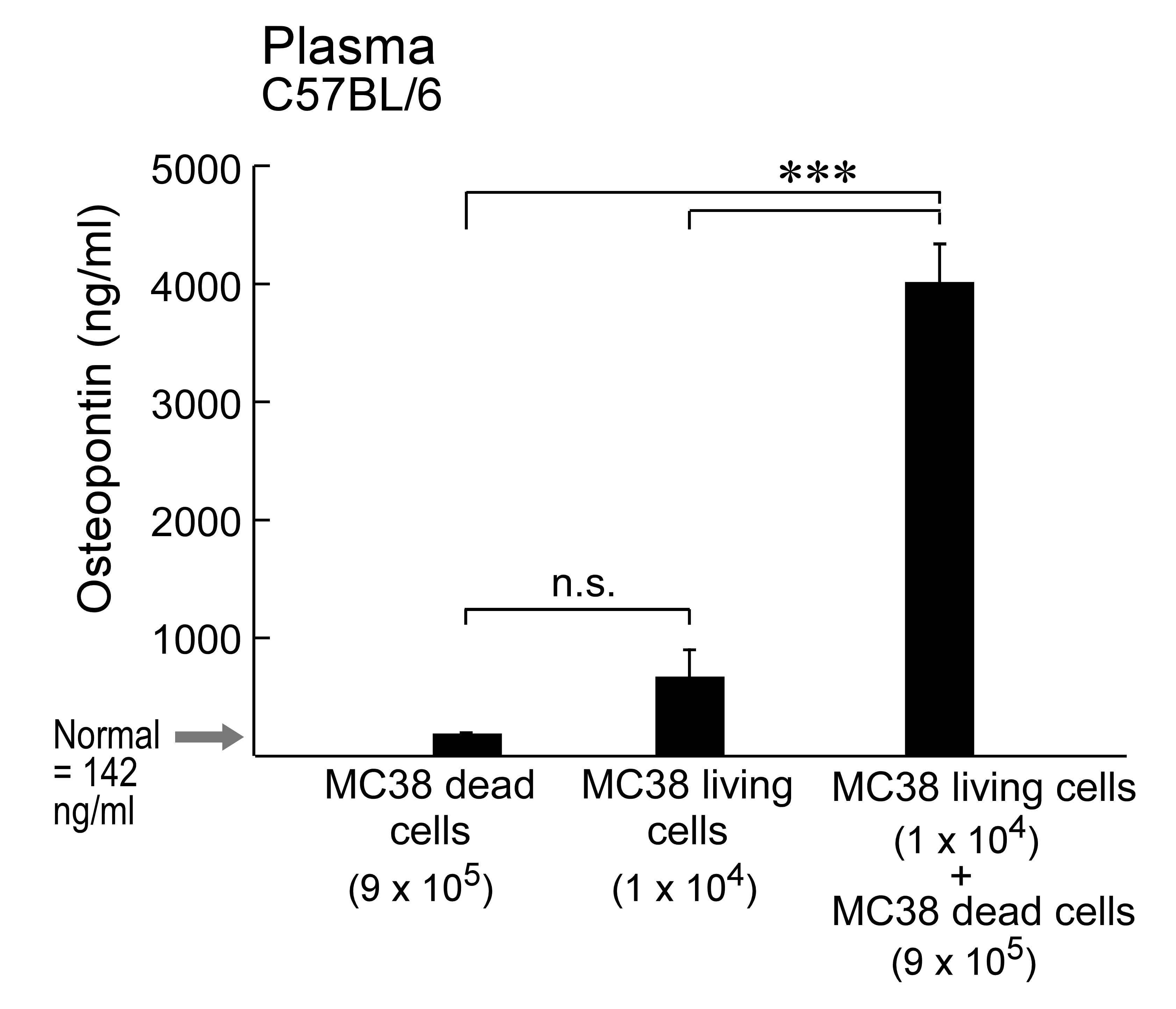

Supplement: Supplementary file 3 [file fj.201800019RR.sf3.tif]
