# Supplementary material for: Chemotherapy-generated cell debris stimulates colon carcinoma tumor growth via osteopontin
Source: FASEB J. 2018 Jun 29;33(1):114–25. doi: 10.1096/fj.201800019RR (PMC6355061; doi:10.1096/fj.201800019RR)
Supplement: Supplementary file 4 [file fj.201800019RR.sd1.docx]

**Supplemental Figure 1.**

**(A)** CT26 tumors (1x10^5^ living cells) treated systemically with 5-FU (30 mg/kg q 3 days). Treatment initiated once tumors reached 100-200 mm^3^. n=5 mice/group. **(B)** CT26 tumors (1x10^5^ living cells) treated systemically with 5-FU (30 mg/kg q 3 days) starting day of injection. n=5 mice/group. **(C)** Flow cytometry analysis assessing apoptotic (annexin V+ propidium iodide (PI)-; bottom right quadrant), necrotic (annexin V-PI+; upper left quadrant), and late apoptotic/necrotic (annexin V+ PI+; upper right quadrant) cell debris in size-matched primary subcutaneous CT26 tumors (5x10^5^ living cells) isolated from Balb/c mice treated systemically with 30 mg/kg 5-FU q 3 days or control. n=4-5 mice/group **(D)** Flow cytometry analysis of cell death in the floating populations of 5-FU-treated CT26, MC38, and RKO via annexin v/propidium iodide (PI) staining. n=3/group. **(E)** Percent survival of mice co-injected orthotopically into the wall of the cecum with 5-FU-generated CT26 dead cells (9×10^4^) and/or CT26 living cells (1×10^3^). Fisher exact test indicated shortened survival of mice injected with a combination of dead and living cells. n=5 mice/group. Data are presented as the mean ± SEM. Two-tailed Student’s t-test for final tumor measurements were used throughout unless specified; *p<0.05, **p<0.01, ***p<0.001.

**Supplemental Figure 2.**

ELISA quantification of murine OPN in conditioned medium from **(A)** CT26 tumor cells exposed to 5-FU-generated CT26 dead cells vs. tumor cells or dead cells alone, or **(B)** MC38 tumor cells exposed to 5-FU-generated MC38 dead cells vs. tumor cells or dead cells alone. n=3/group **(C)** ELISA quantification of human OPN in conditioned medium from RKO tumor cells exposed to 5-FU-generated RKO dead cells vs. tumor cells or dead cells alone. n=3-6/group. **(D)** CT26 tumors (1x10^4^ living cells) treated systemically with 5-FU (30 mg/kg q 3 days) starting on day of injection. n=5 mice/group. **(E)** ELISA quantification of murine OPN in plasma collected from mice injected with 1x10^4^ CT26 treated systemically with 5-FU vs. control. n= 5/group. **(F)** ELISA quantification of murine OPN in plasma collected from non-tumor bearing mice treated systemically with 5-FU vs. control. n= 5/group. **(G)** Viability of MS1 mouse endothelial cells treated with conditioned medium from MC38 pretreated with control, 3 µg IgG/mL, or 3 µg anti-OPN Ab/mL and exposed to 5-FU-generated MC38 dead cells. n=12/group. **(H)** Paraffin-embedded sections of CT26 tumors (1x10^4^ living cells) treated systemically with 5-FU vs control stained with mouse CD31 (brown) counterstained with hematoxylin (blue). Scale bar = 100 µm. Vessel quantification represented as mean ± SD. n= 4-5/group. Data are presented as the mean ± SEM unless specified. Two-tailed Student’s t-test was used throughout; n.s. = not significant, *p<0.05, **p<0.01, ***p<0.001.

**Supplemental Figure 3.**

ELISA quantification of murine OPN in plasma collected from mice injected with 9x10^5^ dead MC38, 1x10^4^ living MC38, or the combination. n= 5/group. Data are presented as the mean ± SEM. Two-tailed Student’s t-test was used; n.s. = not significant, ***p<0.001.
